# Retrosternal Goitre: Anatomical Aspects and Technical Notes

**DOI:** 10.3390/medicina58030349

**Published:** 2022-02-25

**Authors:** Enrico Battistella, Luca Pomba, Gisella Sidoti, Chiara Vignotto, Antonio Toniato

**Affiliations:** Endocrine Surgery Unit, Department of Surgery, Veneto Institute of Oncology, IOV-IRCCS, Via Gattamelata 64, 35128 Padua, Italy; luca.pomba@iov.veneto.it (L.P.); gisella.sidoti@iov.veneto.it (G.S.); chiara.vignotto@iov.veneto.it (C.V.); antonio.toniato@iov.veneto.it (A.T.)

**Keywords:** thyroid surgery, intrathoracic goitre, sternotomy, cervicotomy, morbidity

## Abstract

*Background and Objectives:* surgery for substernal goitre is still debated in the literature, due to the wide range of surgical options. This article outlines the findings of our extensive experiences, which include 264 cases of patients with “goitre plongeant“, and compares postoperative complications, despite surgical approaches. *Material and Methods*: preoperative planning and anatomical landmarks are described to determine the potential need of a combined approach. The surgical procedure is described, along with some stratagems, to ensure that the operation is completed safely. A statistical analysis of complications and the length of stay, with a comparison of cervicotomy and combined access, was performed using the Pearson chi-square significance test. *Results:* 264 patients underwent thyroid surgery for substernal goitre. The Kocher incision was the surgical approach chosen in 256 patients (96.6%), while an accessory incision was performed in 8 patients (3.4%). The necessity to use a two-fold surgical access was linked to a higher rate of postoperative complications (*p*-value < 0.01). The average length of stay (LOS) for cervicotomy was 2 days (1–3 days), while the average LOS was 5 days (4–7 days) (*p*-value = n.s.) for combined access. *Conclusions:* cervicotomy should be the gold standard technique for exploring intrathoracic goitre with a digital dissection, which, in almost all cases, enables the externalization of the mediastinal portion associated. Sternotomy is related to a higher rate of complications, so it should be performed only in selected cases. Management in large-volume centres may be more appropriate.

## 1. Introduction

Thyroid tumefactions that grow in the mediastinum are defined as intrathoracic goitres (retrosternal and intrathoracic are terms used interchangeably in the published literature) when the mediastinal portion is more represented than the cervical one and they overrun the thoracic inlet by two fingers below, or by at least 4 cm [1,2]. The prevalence reported in the literature is highly variable (0.1–21%) with a 10% average; the incidence occurs in about 1/5000 people and, as in other endocrine diseases, is more common in females than in males (F:M = 4:1) [3,4]. Retrosternal goitre can be divided into two types: “plongeant”, when the parenchymal tissue is connected to the thyroid gland and shares the vascularization (95–98% of cases), and autonomous (2–5% of cases), when this connection is not present and may be considered a mediastinal neoformation [5].

“Goitre plongeant” is technically challenging for surgeons due to the difficulties of mediastinum surgery. The mediastinum is usually divided into an anteroposterior direction by two virtual frontal planes. The first one is tangent to the anterior surface of the pericardium and great vessels, and the second is tangent to the anterior surface of the vertebrae. The middle compartment contains all of the vital structures and is significant in the surgical management of retrosternal goitre.

The use of prophylaxis with iodised salt and a greater use of the surgical approach reduce the incidence of large-sized goitres, although they are still found because of the conservative treatment preferred by endocrinologists due to the disease’s benign and asymptomatic nature [6].

This article describes our approach to the disease, from diagnosis to surgical treatment, providing observations on the technical surgery of intrathoracic goitre related to the sites of thyroidal mass. Furthermore, we performed a statistical analysis comparing post-operative complications based on the surgical approach.

## 2. Materials and Methods

Demographic and clinical data of all patients who underwent surgery for intrathoracic goitre at our tertiary referral centre from January 1997 to December 2021 were retrospectively entered into a computerised endocrine surgery registry. All procedures were performed by a single extensively trained endocrine surgery team.

The following definition of intrathoracic goitre was used in our series: thyroid tissue located at least 4 cm below the thoracic inlet, using the classification by Eschapasse and Merlier, as “…*les goitres qui, en position opératoire, descendent au minimum de deux travers de doigt dessous de l’orifice supérieur du thorax*” [2,7].

All patients had a preoperative neck ultrasound (US) and chest X-ray. An additional diagnosis included neck/chest computed tomography (CT) or magnetic resonance imaging to determine the optimal surgical technique [8,9,10]. Furthermore, all patients were evaluated by an otolaryngologist by means of a laryngo-tracheoscopy. Patients undergoing a possible cervico-sternotomy had lung function testing and bronchoscopies (45 patients, 17%).

The indication for surgery and the choice of surgical technique were discussed in a multidisciplinary meeting, involving at least one radiologist, a nuclear medicine specialist, an endocrinologist, an endocrine surgeon, and a pathologist.

Surgery always started with a Kocher incision on the imaginary collarbone line for intrathoracic goitre, at a lower height than the classic cervical approach. Based on the preoperative and perioperative findings, a sternal split or another access was performed when the intrathoracic part could not be safely delivered.

A five-year follow-up was conducted with surgical visits three, six, and twelve months after surgery and then once a year thereafter.

Statistical analysis: the presence of a relationship between the surgical approach, complication rates, and the length of stay was analysed using the Pearson chi-square significance test.

## 3. Results

Since 1997, our department has performed thyroid surgery on 24,876 patients.

Our study included 264 patients diagnosed with intrathoracic goitre (M:F = 1:4). The average age at the time of surgery was 54 years (range 15–86 years).

Intrathoracic goitre was diagnosed in 121 patients (45.8%) owing to the appearance of a cervical mass. Another 25 patients reported dyspnoea and coughing fits caused by compressive symptoms on the trachea (9.5%), 20 patients were diagnosed with hyperthyroidism (7.6%), 8 patients with dysphonia (3%), 7 patients presented dysphagia (2.6%), 5 patients underwent emergency surgery for acute respiratory failure due to tracheal obstruction (1.9%), 4 patients presented vein compression (1.5%), and 74 patients (28.1%) discovered retrosternal goitre incidentally.

Since 2007, preoperative CT or MRI has been used to determine the boundaries of the intrathoracic goitre and to define the more appropriate access in 167 patients (63.2%) (Table 1).

The Kocher incision was the surgical approach chosen in 256 patients (96.6%), including prevascular, retrovascular, and autonomous goitres.

An accessory incision was performed in 8 patients (3.4%): 5 cervico-mediastinal goitres, 3 mediastinal goitres, 2 anterolateral thoracotomies, and 6 partial sternotomies. The anterolateral thoracotomies were performed in the first period of our experience for an autonomous goitre and for a left intrathoracic goitre strictly connected to the sovra-aortic vessels. A partial sternotomy was performed for four suspected neoplastic degenerations and when the digital manoeuvre could not be performed safely in two other cases.

**Figure 1:**
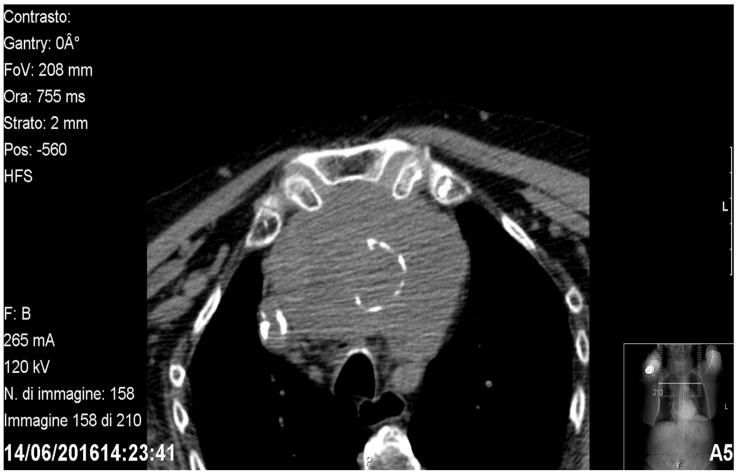
Axial CT showing a prevascular retrosternal goitre.

**Figure 2:**
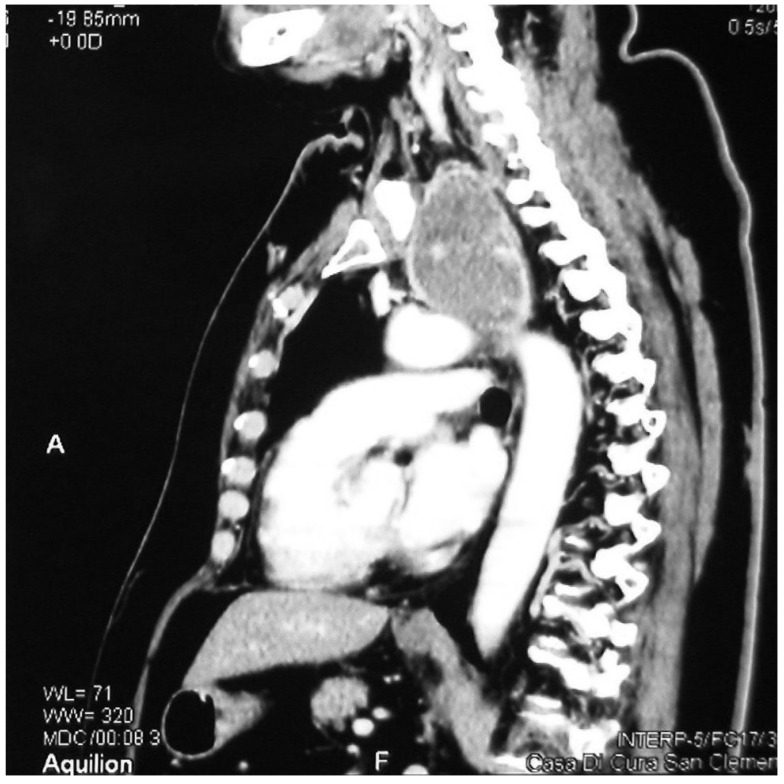
Sagittal CT showing a retrovascular retrosternal goitre.

A total of 173 cervico-mediastinal goitres (67.6%) resulted from a prevascular localization and 83 (32.4%) were in retrovascular positions (Figure 1 and Figure 2). There were 8 autonomous goitres (3%): 7 prevascular and 1 retrovascular.

Total thyroidectomy was performed in 135 patients (51%), near-total thyroidectomy was performed in 95 patients (36%), thyroid lobectomy in 31 patients (11%), whereas only the mass was removed in 3 patients (1%). Suspected thyroid nodules in 10 patients were treated with total thyroidectomy.

Postoperative complications included: 9 (3.4%) patients who manifested postoperative hypocalcaemia, 7 patients (2.6%) who reported transient recurrent laryngeal nerve injury, 6 cases were monolateral, and 1 case was bilateral, requiring the positioning of a temporary tracheostomy, 2 patients (0.75%) presented postoperative bleeding, and 1 patient died (0.37%) as a result of acute respiratory failure caused by tracheomalacia.

An increase in postoperative complications necessitated the use of double surgical access: 4/8 patients versus 16/256 patients in cases where only a cervical approach was used. Our statistical analyses comparing post-operative complications in the two surgical options resulted significant (*p*-value < 0.01), but we should mention that, in our case, only the most difficult cases were treated with a combined approach.

As a result of histological findings, struma was diagnosed in 254 cases (96.2%) and carcinoma in 10 cases (3.8%).

The average length of stay (LOS) for cervicotomy was 2 days (1–3 days), while the average LOS for combined access was 5 days (4–7 days) (*p*-value = n.s.) (Table 2).

## 4. Discussion

Substernal goitre can be a challenging disease due to the mediastinal extension that brings it into close contact with vital structures. The advancement of diagnostic tools, such as CT or MRI, led to an increase in cases operated on in our department. The first period of surgery, from 1997 to 2007, included 60 patients (22.7%), while the second period, 2008–2021, included 2044 patients (77.3%).

According to the literature, the presence of symptomatic retrosternal goitre and possible dimensional increase in asymptomatic young patients are both indications for surgery [11,12]. The dimensional increase could compress the oesophagus, trachea, recurrent laryngeal nerves, and vessels, necessitating an emergency surgical procedure. Elective surgery serves a therapeutic and preventive purpose, preventing neoplasm degeneration. Radioactive iodine treatment cannot achieve control of the disease and can also result in an acute inflammatory process that exacerbates clinical compression, potentially threatening the patient’s airway [3,4].

The starting point of the surgical procedure is the asportation of the largest part of the mass. If just one lobe is involved and the other is normal, surgery should consist of a hemi-thyroidectomy, minimizing the risk of complications while relieving symptoms of concern. If the dissection of the goitre from the laryngeal nerve or the vascular plane is not well-defined, a small portion of thyroid tissue should be left to avoid damage. Roman et al. identified key reasons for special attention to be paid in the preoperative laryngeal examination: the presence of vocal cord paralysis (VCP) may be clinically evident in the absence of dysphonic changes, and the presence of VCP influences the patient’s consent and counselling on the risks of potential surgery [10].

The choice of the surgical approach in a retrosternal goitre is a debated topic. In the literature, several authors claim that an isolated cervicotomy can completely remove the intrathoracic thyroid tissue [13,14,15]. According to Sormaz et al., a CT-volume in the mediastinal portion of a thyroidal mass greater than 162 cm^3^ and a craniocaudal length below the thoracic inlet greater than 66 mm are significantly associated with the need for an extra-cervical approach [16]. Furthermore, Huins et al. performed a review that included 34 studies and developed a three-grade system based on the substernal extension of the thyroidal mass: the first grade above the aortic arch, the second grade at the level between the aortic arch and the pericardium, and the third grade extending below the right atrium. They concluded that the sternal split, or sternotomy, is safer for thyroid glands in the second and third grades. Simo et al. reported four significant anatomical landmarks to detect high-risk patients, including the involvement of the carina of the trachea, the arch of the aorta, the pleura of the lungs bilaterally, and the oesophagus [17].

Cervicotomy was the gold standard in our series, and surgical success was achieved in 96.6% of cases. We also used it as the starting point to explore the goitre and its boundaries and to proceed with the dislocation of the mass using the connection between the thyroid lobe and the mediastinal portion. This is made feasible by the process of migration of the mediastinal portion, which maintains the vascularization shared with the cervical segment. Furthermore, we should mention that cervicotomy presented a lower rate of post-surgery complications than combined access (*p*-value < 0.01) and a shorter length of stay (*p*-value = n.s.). Some authors claim that bleeding is a life-threatening condition that is difficult to control only with cervical access [18]. We have only had two cases of post-operative bleeding, one in each surgical approach. We avoided this complication through accurate preoperative planning of the surgical technique and by ligating the inferior thyroid artery near its beginning. Furthermore, most of this bleeding occurred within the first 8 h of surgery and was accompanied by coughing fits. It can lead to laryngeal oedema and potential airway obstruction, including death [17,18].

The success of our surgical strategy is based on some stratagems. First of all, the patient’s position should not be excessively hyper-extended to avoid the contraction of the neck’s ribbon-like muscles, thus causing a difficult dissection. The second important stratagem is to perform a broad and low cervicotomy (on the clavicular line), extending the distal opening of the linea alba to the retrosternal insertion of the pre-thyroid muscles, and their subsequent detachment from the sternocleidomastoid. If necessary, a partial or total section of the pre-thyroid muscles is a useful way to stretch the thyroid loggia and make space for the cleavage. After ligating and dissecting the middle vein (if any, and if accessible), and then the superior peduncle, the strict plane of the extracapsular dissection guarantees a gradual finger dissection. It is recommended that the inferior thyroid artery is ligated close to where it originates at the trunk as a preventive measure to ensure haemostatic control, or in the event that it interferes with the luxation of the sunken portion, and the digital identification of the recurrent laryngeal nerve, which is sometimes displaced posteromedially to the goitre and fused with the capsular plane. At the end, if possible, we usually search for the sub-isthmus trachea and follow it into the mediastinum to locate the goitre’s dissection plane, starting from the side that is less affected. We usually externalise the goitre with the index finger to pull the plongeant portion. If this blind manoeuvre is difficult to achieve, we consider it useless to only proceed with cervical access because it can cause a dangerous vascular lesion. We recommend using combined access or another type of access when it is impossible to reach the lower margin of the plongeant goitre with a digital dissection and when it is impossible to remove the mediastinal portion from the thoracic inlet or to separate it from the mediastinal structures due to an inflammatory or neoplastic process. A high degree of kyphosis, vein stasis due to the compression of the mediastinal vein, and emergency situations are all cases where combined access should be used [3,4].

**Figure 3:**
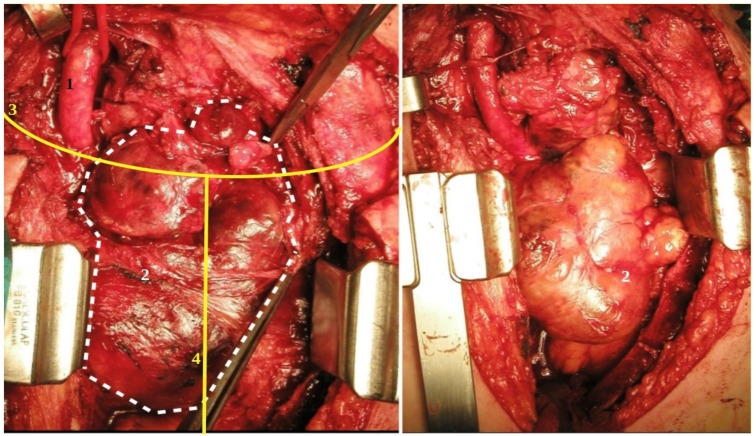
Cervicotomy and sternotomy for endothoracic goitre: ((**1**) common carotid artery; (**2**) endothoracic anterior goitre; (**3**) cervicotomy; (**4**) sternotomy).

Sternotomy enables the removal of the retrosternal goitre, which would otherwise be difficult to explore safely through cervicotomy due to the nature, shape, dimension, site, and boundaries with other mediastinal structures. In accordance with the literature, the gold standard of access in this situation is partial or total sternotomy that we performed in 6 patients (Figure 3). The indications are goitre plongeants with large dimensions, those that are symptomatic, prevascular, or retrovascular on the left side, those that are difficult to remove, and those with neoplastic degeneration in the mediastinal portion [19,20].

Combined access in the form of thoracotomy and cervicotomy is rare (only two cases in our initial experience). Anterior thoracotomy is easy to perform, but it is difficult to obtain a view of the right recurrent laryngeal nerve. Thoracotomy is not recommended if the mediastinal portion is developed on the left side, due to the presence of the sovra-aortic vessels. Posterolateral thoracotomy prolongs surgical time due to the change in position. Indications include a large posterior goitre on the right side, with suspected malignant degeneration, and thoracic structural adhesions [21].

According to the literature, the primary target is the removal of mechanical obstacles and the prevention of future compressive symptoms, especially in young people [22]. This pathology should also be treated in a referral centre in order to ensure optimal results and low morbidity rates and complications; ability, experience, profile, availability of experienced personnel, and proper materials must not be underestimated [23]. The surgical team’s experience is another crucial factor in ensuring surgical success, despite the obstacles associated with the goitre’s dimension and location.

## 5. Conclusions

In our opinion, the surgical approach should be determined by whether the disease is mono or bilateral, and by taking into account the cytomorphology of the disease and the patient’s perioperative risk based on comorbidity. Cervicotomy should be the gold standard technique to explore intrathoracic goitre with a digital dissection that permits externalization of the mediastinal portion in almost all cases. If complementary access is necessary, partial sternotomy is the most indicated. Furthermore, our statistical analysis revealed a significant lower rate of postoperative complications using cervicotomy access rather than combined access. Preventing perioperative complications is the primary goal of the appropriate surgical approach. Management in large-volume centres may be more appropriate, due to the availability of more experienced personnel and better equipment.

## Figures and Tables

**Table 1:** General data of patients included in the study.

Diagnosis	N° Patients = 264 (Male:Female = 53:211)	%
Appearance of cervical mass	121 patients	45.8%
Dyspnoea and cough	25 patients	9.5%
Hyperthyroidism	20 patients	7.6%
Dysphonia	8 patients	3%
Dysphagia	7 patients	2.6%
Acute Respiratory failure	5 patients	1.9%
Vein compression	4 patients	1.5%
Incidentaloma	74 patients	28.1%
**Pre-operatively imaging**		
Neck ultrasound	264 patients	100%
Chest X-ray	264 patients	100%
Neck and chest CT	115 patients	70%
Neck and chest MRI	52 patients	30%

**Table 2:** Results of the clinical series and statistical analyses.

No. Patients = 264	Kocher Incision	Kocher Incision and Accessory Incision	Statistical Analysis
**Surgery**			
Total thyroidectomy	128 cases	7 cases	
Near-total thyroidectomy	94 cases	1 case	
Thyroid lobectomy	31 cases	/	
Mass removal	3 cases	/	
**Post-operative complications**	**16/256 patients**	**4/8 patients**	***p*** **-value < 0.01**
Hypocalcaemia	10 cases	/	
Monolateral transient recurrent laryngeal nerve injury	5 cases	1 case	
Bilateral transient recurrent laryngeal nerve injury	/	1 case	
Post-operative bleeding	1 case	1 case	
Death	/	1 case	
**Histological report**			
Struma (254 cases, 96.2%)	252 cases	2 cases	
Thyroid carcinoma (10 cases, 3.8%)	4 cases	6 cases	
**Length of stay**	**2 days (1–3 days)**	**5 days (4–7 days)**	***p*-value = n.s.**

## Data Availability

Data could be found in the patients’ medical records.
